# Quantifying extracellular matrix turnover in human lung scaffold cultures

**DOI:** 10.1038/s41598-018-23702-x

**Published:** 2018-04-03

**Authors:** Oskar Rosmark, Emma Åhrman, Catharina Müller, Linda Elowsson Rendin, Leif Eriksson, Anders Malmström, Oskar Hallgren, Anna-Karin Larsson-Callerfelt, Gunilla Westergren-Thorsson, Johan Malmström

**Affiliations:** 1 Lung Biology, Department Experimental Medical Science, Lund University, Lund, Sweden; 2 Division of Infection Medicine, Department Clinical Sciences, Lund University, Lund, Sweden; 3 Department Respiratory Medicine and Allergology, Lund University, Lund, Sweden

## Abstract

Remodelling of the extracellular matrix is accomplished by altering the balance between matrix macromolecule production and degradation. However, it is not well understood how cells balance production of new matrix molecules and degradation of existing ones during tissue remodelling and regeneration. In this study, we used decellularized lung scaffolds repopulated with allogenic lung fibroblasts cultured with stable isotope labelled amino acids to quantify the balance between matrix production and degradation at a proteome-wide scale. Specific temporal dynamics of different matrisome proteins were found to correspond to the proliferative activity of the repopulating cells and the degree of extracellular deposition. The remodeling of the scaffold was characterized by an initial phase with cell proliferation and high production of cell adhesion proteins such as emilin-1 and fibronectin. Extended culture time resulted in increased levels of core matrisome proteins. In a comparison with monolayer cultures on plastic, culture in lung scaffolds lead to a pronounced accumulation of proteoglycans, such as versican and decorin, resulting in regeneration of an extracellular matrix with greater resemblance to native lung tissue compared to standard monolayer cultures. Collectively, the study presents a promising technique for increasing the understanding of cell- extracellular matrix interactions under healthy and diseased conditions.

## Introduction

The human lung is continuously challenged with damaging agents such as microbes, allergens, polluted air and smoke. Consequently, there is an ongoing process of repair and regeneration to maintain tissue homeostasis. An inadequate or imbalanced response to tissue damage plays a part in the development of a variety of conditions such as asthma, idiopathic pulmonary fibrosis (IPF) and chronic obstructive pulmonary disease (COPD), responsible for considerable morbidity and mortality^1–3^. COPD, for example, a major cause of morbidity and mortality^4^, is representing the third leading cause of death worldwide^5^ and is lacking preventive or curative treatment. A common denominator in these conditions is an extensive reorganization of the extracellular matrix (ECM)^2,3,6^. In these disease states the tightly controlled interplay between ECM and ECM modifying cells is distorted, leading to pathological tissue remodelling. ECM remodelling in the lung involves structures such as blood vessels, bronchi, alveoli, basal membranes and surrounding ECM^6^. The balance between matrix production and breakdown is controlled by inherent cellular properties, by the structure and composition of the ECM itself and by the molecular interplay between the cells and ECM.

We have used the definition of ECM and ECM-associated proteins, commonly referred to as the matrisome, as described by Naba *et al*.^7^. The ECM proteins of the matrisome can be classified as collagens, ECM glycoproteins or proteoglycans; these three protein groups constitute the core matrisome. The ECM associated matrisome proteins are in turn subdivided into three groups; secreted factors, ECM-affiliated proteins and ECM regulators.^7,8^. The matrisome proteins produced in lung tissue fulfil many different functions from generating tissue stability to containing information-rich macromolecules governing the properties of lung tissue^6,9–12^. To generate and maintain the complex tissue architecture, the lung requires several cell types such as fibroblasts, epithelial, endothelial and immune cells. These cells interact actively with the surrounding ECM, which in turn exerts powerful influences on the resident cells^6,13^.

Tissue engineering using acellular biological scaffold obtained through decellularization of tissues or organs may in the future help alleviate the shortage of donor organs for transplantation^14–16^. Improved understanding of the interaction of tissue resident cells and ECM may serve to improve current tissue engineering efforts and help to find new strategies to address diseases such as COPD and IPF. Acellular scaffolds contain tissue-specific ECM capable of maintaining functional cellular phenotypes and has been shown to support recellularization with tissue resident cells in several organs such as heart, liver, kidney and pancreas^17–24^. During the recellularization process, the resident cells remodel the scaffold, which in turn influences cellular behaviour. A proteomics based strategy to study this remodelling process using stable isotope labelling with amino acids in cell culture (SILAC) has recently been described by Li *et al*.^25^. Using decellularized vocal folds it was shown that immortalized vocal fold fibroblasts cultured with isotopically labelled amino acids produced heavy labelled versions of all newly synthesized proteins. These heavy labelled, newly synthesized proteins were distinguishable from the resident proteins found in the acellular scaffolds using mass spectrometry (MS)^25^. Quantitative comparison of the heavy and light proteins provides a direct measurement of protein turnover in these repopulated scaffolds and represents a promising and emerging technology in tissue engineering.

In this communication, we have adapted the SILAC mass spectrometry strategy to investigate matrisome turnover by primary human lung fibroblasts in acellular scaffolds obtained from healthy human lung tissues. Our results outline important differences in ECM deposition by fibroblast in acellular scaffolds as compared to growth in traditional monolayers on plastic surfaces. The outlined results reveal missing and important knowledge about the turnover of ECM in repopulated biological scaffolds and the complex interplay between ECM and tissue resident cells.

## Materials and Methods

Lung explant tissue from healthy organ donors (*n* = 2) with no history of lung disease were included. Lungs were to be used for transplantation but could instead be included in this study as no matched recipient could be identified. Written informed consent was obtained from their closest relatives. Surgery was performed at Sahlgrenska University hospital, Gothenburg. This study was approved by the Swedish Research Ethical Committee in Lund (2008/413) and all experimental protocols were carried out in accordance with guidelines approved by the ethical committee. No organs or tissues were procured from prisoners.

### Primary parenchymal lung fibroblasts

Primary human lung fibroblasts from parenchymal lung tissue were isolated from one donor lung as previously described^26^. Primary fibroblasts were expanded in Dulbecco’s Modified Eagle Medium (DMEM, Sigma Aldrich, St Louis, MO, US) supplemented with 10% foetal clone serum (FCIII, Thermo Scientific, Waltham, MA, US), 1% L-glutamine, 1% penicillin-streptomycin (PEST), 0.5% gentamicin and 5 µg/ml amphotericin B (all from Gibco BRL, Paisley, UK) at 37 °C, 10% CO_2_. The primary fibroblasts were expanded until passage 4–5 before seeding into either decellularized lung tissue slices or plastic tissue culture dishes.

### Preparation of decellularized lung scaffolds

Cubes of peripheral lung tissue of approximately 8 mm^3^ and with the pleura kept on one side were dissected from a different donor lung than the one from which the fibroblasts were isolated. The tissue cubes were placed in a petri dish cooled with liquid nitrogen, snap frozen and stored at −80 °C. Tissue cubes were sectioned at 350 µm in a HM-560 cryostat (Microm, Heidelberg, Germany). An antifreeze cryoprotectant solution (30% v/v glycerol and 30% v/v ethylene glycol in 0.1 M sodium phosphate buffer) was applied to the tissue to facilitate sectioning without disrupting tissue architecture. Newly cut sections were placed in chilled PBS for rapid thawing. Slices were then treated with decellularization solution consisting of 8 mM CHAPS (ICN biomedicals Inc., Aurora, OH, US), 1 M NaCl, and 25 mM EDTA in PBS^27–29^. The treatment was performed on a rocking table with 1 ml/slice of decellularization solution, which was changed six times over a period of four hours at room temperature. Treated slices were then extensively rinsed in benzonase working buffer comprised of 20 mM Tris-HCl, 2 mM Mg^2+^ and 20 mM NaCl at pH 8, and treated with 1 ml/slice of 90 U/ml of benzonase nuclease (Sigma-Aldrich) for 30 min at 37 °C. Decellularized slices were rinsed in PBS and stored overnight at +4 °C in PBS containing 1% PEST, 0.5% gentamicin and 5 µg/ml amphotericin B. Decellularized slices were fixed for histology and SEM or frozen at −80 °C until MS or DNA analysis. DNA content of decellularized and native tissue slices was quantified with Quant-iT PicoGreen dsDNA assay kit (Invitrogen) according to the manufacturer’s instructions. Slices for DNA analysis were lyophilized (Freezone 6, Labconco Co., Kansas City, MO, US), weighed, diluted in TE-buffer, homogenized with a FastPrep FP120 bead beater (Qbiogene, Illkirch, France) and incubated with the Quant-iT PicoGreen reagent. Fluorescence measurements were made at 520 nm with excitation at 485 nm.

### Cell Culture

Primary human lung fibroblasts were cultured with SILAC™ Protein ID & Quantitation Media Kit, with Lysine and D-MEM-Flex (SILAC-DMEM, Cat. MS10030, Life Technologies, Carlsbad, CA, US) prepared according to the manufacturer’s instructions containing 4,5 g/l glucose, 10% dialyzed fetal bovine serum and with the exclusion of phenol red. Cells were cultured in medium containing either normal L-lysine (Lys = light) or with [U-13C6]-L-lysine (Lys-6 = heavy). Decellularized lung tissue slices were incubated with SILAC-DMEM for 1 h at 37 °C, 8% CO_2_ before seeding with fibroblasts by replacing the medium with 1 ml of medium containing 5 × 10^4^ cells. 24-well suspension culture plates with a hydrophobic growth surface (Sarstedt, Nümbrecht, Germany) with one seeded slice per well were incubated on an orbital shaker (19 mm orbit, 100 rpm) for the first 24 h, to provide the cells with the opportunity to adhere to favourable sites in the scaffolds. However, we expect that most cells will attach within a few hours. Media were changed every 72 h and at the first media change the slices were lifted into new culture plates. Control cultures of fibroblasts were seeded into standard tissue culture plates 5,5 × 10^4^ in 12-well or 1,5 × 10^5^ in 6-well plates (Cell+, Sarstedt), first 24 h of culture was performed on an orbital shaker, Lys-containing media were used and changed every 72 h. Samples were collected after 6, 9, 12 and 25 days of culture.

### Electron microscopy

Lung slices for scanning electron microscopy (SEM) were fixed in buffer containing 0.1 M Sorensen´s phosphate buffer pH 7.4, 1.5% formaldehyde and 1.5% glutaraldehyde at RT for 2 hours. After fixation, the samples were washed twice in 0.1 M Sorensen´s phosphate buffer pH 7.4, before being dehydrated in a graded series of ethanol (50%, 70%, 80%, 90% and twice in 100%). Samples were critical point dried before being mounted and examined in a Jeol JSM-7800F FEG-SEM at Lund University Bioimaging Center.

### Morphology and histology

Lung slices for histology were fixed in 4% formaldehyde for 30 min, dehydrated, embedded in paraffin and sectioned with a thickness of 4 µm. Hematoxylin and Eosin (HE) and Verhoeff van Gieson (VVG, HT25A, Elastic Stain Kit, Sigma Aldrich) staining was used to assess overall morphology and elastic fibers, respectively. Apoptotic cells were detected by labeling of exposed 3′-OH ends of DNA fragments using ApopTag® Red *In Situ* Apoptosis Detection Kit (TUNEL, S7165, Merck Millipore, Darmstadt, Germany) with nuclear counterstaining using 4′,6-diamidino-2-phenylindole (DAPI). Collagen type IV and Ki-67: Heat mediated antigen retrieval was followed by incubation with primary antibodies for 1.5 h, incubation with secondary antibodies for 45 min and DAPI counterstaining (Supplementary Table S1). Negative controls were performed by omitting the primary antibodies. Quantification of TUNEL and Ki-67 stained cells was performed using the Fiji software (v1.50i, National Institutes of Health).

Immunohistochemistry (IHC) for versican, galectin-3 and decorin was performed using the EnVision Dual Link System (K4065, Dako, Glostrup, Denmark) according to manufacturer’s instructions. Antigen retrieval, antibody dilution and secondary antibodies are specified in Supplementary Table S1. Secondary antibodies coupled to horseradish peroxidase produced brown precipitates. Sections were counterstained with Mayer’s Hematoxylin for visualization of nuclei. Validation of antibodies was performed in previous studies^30–32^, unspecific staining was assessed by omitting the primary antibodies. Images of entire stained sections were obtained with a VS120 virtual microscopy slide scanning system (Olympus, Tokyo, Japan). Selected images were obtained using the OlyVIA software 2.8 (Olympus). The immunofluorescence samples were scanned with the same acquisition settings, and pictures were adjusted with the same histogram modifications, for each respective staining.

### Protein extraction from tissue and cell cultures

A consecutive extraction strategy was used for in-depth protein extraction from lung tissue slices, sample proteins were fractionated into soluble fractions, SDS released fractions and ECM-enriched fractions^33^. All tissue slices (native, decellularized or repopulated with heavy labeled cells) were lyophilized, diluted in PBS (10 mM phosphate, 2.7 mM KCl, 137 mM NaCl, pH 7.4), mixed with 0.1 mm silica beads (BiospecProducts, Bartlesville, OK, USA) and homogenized using a FASTprep-96 instrument (1,600 g, 180 s) (MP Biomedicals, Santa Ana, CA, US). Samples were centrifuged (14,000 g, 1 min) and the supernatants containing the soluble protein fractions were recovered. Subsequently proteins were extracted by incubation for 5 min at 95 °C in 0,15 M NaCl, 0,050 M Tris-HCl, pH 7.6 (TBS) with 2% (w/v) SDS added. After centrifugation (14,000 g, 10 min), the supernatants were recovered containing the SDS released protein fractions. Lastly the samples were clarified from SDS using a short preparative SDS-PAGE run at 200 V for 10 min, carefully recovered from the gel wells and digested using an in-solution protocol^34^. The protein concentrations of the soluble and the SDS released fractions were determined using Pierce BCA Protein Assay Kit (Thermo Scientific, Waltham, MA, USA), prior to in-solution digestion. Fibroblast monolayers cultured on standard culture plastic dishes were rinsed with PBS and detached by freezing at −20 °C, thawing, immersion in dH_2_O and abrasion by a rubber cell scraper before MS sample preparation.

### Mass spectrometry sample preparations

Samples were lyophilized and resuspended in 100 mM ammonium bicarbonate with 8 M urea before reduction with 5 mM tris (2-carboxyethyl) phosphine (TCEP), 30 min at 37 °C at 850 rpm, alkylation with 10 mM iodoacetamide (IAA) 45 min at room-temperature at 850 rpm, and then diluted with ammonium bicarbonate to a urea concentration below 2 M. Digestions were first performed with Lys-C (1:100 w/w) for 2 h, 850 rpm at 37 °C followed by overnight digestion with trypsin, 850 rpm at 37 °C (1:100 w/w). Digestion was stopped by addition of formic acid to 1% concentration. Samples from the tissue slices of the soluble and the ECM-enriched fraction were desalted using C18 columns reversed-phase spin columns (Harvard Apparatus, Holliston, MA, USA). The SDS released sample fractions were cleaned from SDS using the protein SP3 beads protocol as described in^34^. Cell culture samples were desalted using SOLAµ™ Solid Phase Extraction (SPE) Plates (Thermo Scientific, Waltham, MA, USA), according to manufacturer’s instructions.

### LC-MS/MS analysis

All samples were desalted and resuspended in 2% acetonitrile, 0.1% formic acid before LC-MS/MS analysis on a Q Exactive Plus mass spectrometer (Thermo Fisher Scientific). Peptide separation was performed on an EASY-nLC 1000 liquid chromatography system (Thermo Fisher Scientific) connected to an EASY-spray column (Thermo Scientific, ID 75 µm × 25 cm). Buffer A (0.1% formic acid) and buffer B (0.1% formic acid in 100% acetonitrile) was used to run a gradient from 5–30% buffer B (0.1% formic acid, 100% acetonitrile) over 60 min, 30–95% buffer B for 5 min, and finally 95% buffer B for 10 min, using a flow rate of 300 nl/min. A full mass scan at mass range 400–1,600 m/z (resolution 70,000 @ 200 m/z) was followed by MS/MS scans (70,000 @ 200 m/z). The top 15 most abundant ions were selected for fragmentation by higher-energy collisional dissociation (HCD). The dynamic exclusion window was set to 20 s and a MS precursor value above 1.7e4 was required for triggering MS/MS scans. The automatic gain control was 1e6 and the ion accumulation time in MS was set to 100 ms and to 60 ms in MS/MS. Three biological replicates were analyzed for each experimental condition except for the 25-day monolayer culture, which had two biological replicates.

### Data analysis

Raw files were analyzed in MaxQuant (version 1.5.3.30). The resulting peak lists were searched in Andromeda against a reviewed UniProt human database (Swiss-prot downloaded 2015-11-17), complemented with the standard MaxQuant contaminant database. Enzyme specificity was set to trypsin and a maximum of two missed cleavages. Precursor mass tolerance was set to 4.5 ppm and fragment ion mass tolerance to 20 ppm. Carbamidomethylation of cysteine was used as fixed modification and methionine oxidation as variable modifications. The false discovery rate (FDR) was set to 0.01 for both peptides and proteins. The mass spectrometry proteomics data have been deposited to the ProteomeXchange Consortium via the PRIDE partner repository^35^ with the dataset identifier PXD007995.

### Statistical analysis

Data analyses were performed using GraphPad Prism V.7.00 (GraphPad Software, San Diego, California, USA). Differences between two and multiple groups were made using the Mann-Whitney test and the Kruskal-Wallis test, respectively. Weighted scatter plots and heat maps were generated using R Studio Version 0.99.903 (RStudio Team (2015). RStudio: Integrated Development for R. RStudio, Inc., Boston, MA, USA).

## Results

### Evaluation of decellularized lung slices

**Figure 1:**
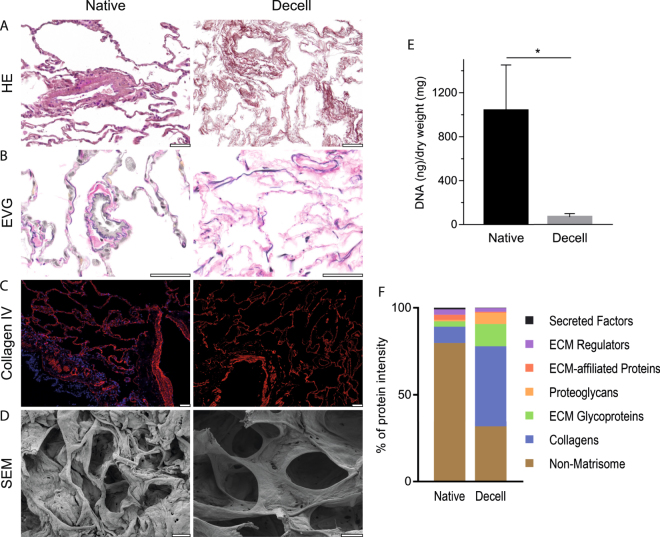
Decellularization produces acellular scaffolds with preserved ECM structure. Decellularized lung slices (Decell) compared to native lung slices (Native). (**A**) HE staining indicating intact morphology and complete removal of cell nuclei (lilac) after decellularization. (**B**) Elastic Van Gieson (EVG) staining with elastic fibers in dark blue. (**C**) Immunofluorescence staining of the basement membrane protein Collagen type IV (red) with nuclear DAPI counterstain (blue). (**D**) Scanning electron microscope (SEM) images of alveolar tissue. Scale bars: 50 µm. (**E**) DNA quantification of native and decellularized lung tissue, error bars show SD. (**F**) Relative distribution of summed protein intensities for respective matrisome or non-matrisome protein group.

Using scaffolds of decellularized lung tissue together with SILAC-MS is a promising alternative for quantitative analysis of ECM turnover, provided that ECM integrity is maintained after decellularization while cell remnants are sufficiently removed to allow for proper repopulation^36^. To produce decellularized lung tissue scaffolds of high quality with maintained ECM integrity, we modified a previously established sample preparation protocol^27^. Lung tissue from an organ donor without lung disease was snap frozen followed by cryosectioning before cells were removed using a combination of hypertonic detergent based decellularization solution and enzymatic DNA degradation. According to the histologic evaluation our decellularization protocol resulted in an even decellularization of the tissue (Fig. 1A and B). As seen with hematoxylin and eosin (HE) staining in Fig. 1A, the overall tissue morphology is well preserved with continuous alveolar septa and patent bronchiolar airways. Eosinophilic staining (pink) is markedly reduced due to removal of cell cytoplasm and hematoxylin staining (lilac) shows absence of cell nuclei from the decellularized tissue. Elastic Van Gieson (EVG) staining demonstrates that the elastic fibers (blue-black) are well preserved both in alveolar septa and in elastic lamina of blood vessels after decellularization (Fig. 1B), in contrast to other studies of decellularized lung tissue^37,38^.

The relatively brief (4 h) exposure to the detergent solution and the use of a zwitterionic detergent (CHAPS) yielded a decellularized tissue with well-preserved basement membranes when evaluated with SEM and immunofluorescent staining for the basement membrane protein collagen type IV. Continuous collagen IV staining was present along alveolar septa and at the basement membranes of airways (Fig. 1C). The SEM images revealed intact alveolar structures in the decellularized tissue with considerably thinner septa compared to the native tissue (Fig. 1D). Similar results regarding the preservation of basement membranes have previously been achieved using the same decellularization solution in lung tissue^27,29^ and in studies using CHAPS for decellularization of vocal folds^25^. Quantification of DNA content using a fluorometric assay showed a 93% reduction of DNA to a concentration of 68 ng/ml dry weight in the decellularized tissues (Fig. 1E) confirmed by the absence of DAPI counterstaining (Fig. 1C). Proteomic evaluation of decellularized lung slices revealed that the samples were dominated by the core matrisome group whereas the cell-associated non-matrisome were reduced from 80% of the proteins in native tissue to 32% after decellularization. (Fig. 1F, summed relative intensities). Comparable levels of non-matrisome residues have been achieved using other decellularization protocols^39,40^. Collectively these results show that decellularized lung slices can be produced from pieces of resected human lung tissue smaller than 1 cm^3^, achieving low levels of residual DNA while preserving morphology with continuous basement membranes.

### Characterization of repopulated lung slices

**Figure 2:**
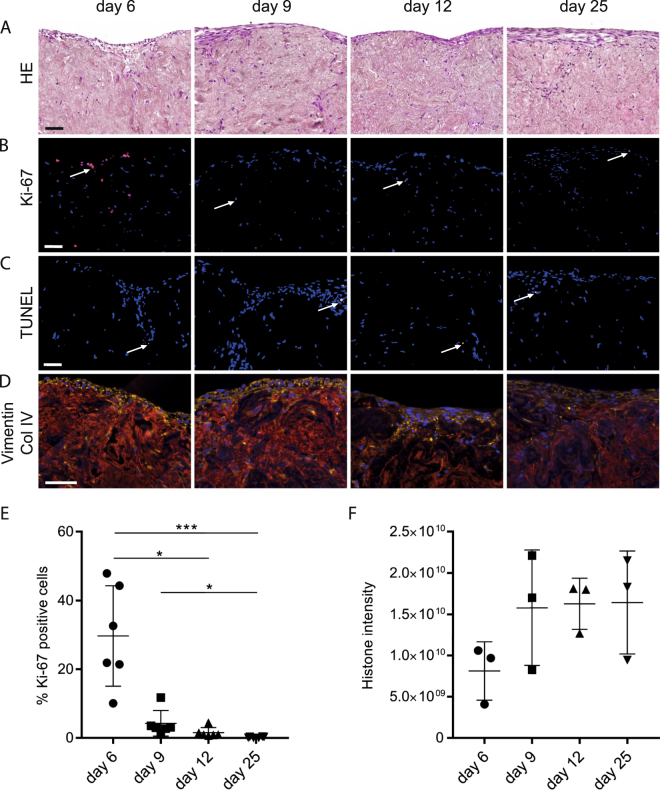
Culture of fibroblasts in decellularized lung scaffolds. Representative images from scaffolds at four different time points; day 6, 9, 12 and 25, are shown, including an outer edge of the scaffold at the top of each image in A–D. (**A**) HE staining of repopulated scaffolds at four time points. Cell nuclei are seen dispersed within the scaffold and lining the edge of the scaffold. (**B**) Ki-67 staining (red) for proliferating cells with nuclear DAPI counterstain (blue), arrows: Ki-67 positive cells. (**C**) TUNEL staining indicating few apoptotic cells (yellow) at each time point, arrows: TUNEL positive cells. (**D**) Staining for cytoskeletal protein Vimentin (yellow) showing the location of cell bodies. The basement membrane protein Collagen type IV (red) visualizes the scaffold, cell nuclei stained with DAPI (blue). Scale bars: 50 µm. (**E**) The percentage of Ki-67 positive cells quantified in six entire tissue sections from two or three individual 3D scaffolds for each time point (error bars show SD). (**F**) Cell density as quantified by the summed histone intensities at all time points, error bars show SD.

To investigate the properties of the decellularized tissue we seeded and incubated the scaffolds in a fibroblast cell suspension for 24 h with continuous agitation, allowing the cells to attach to available surfaces in the scaffold. Control slices were incubated in identical growth medium devoid of any cells. Seeded lung slices were visibly condensed into compact tissue samples until approximately day nine, thereafter there was no visible additional decrease in outer surface area (data not shown). This phenomenon has previously been observed with fibroblasts in lung tissue scaffolds^37^. Histologic evaluation of cultured scaffolds revealed dispersed cells throughout the entire scaffold, although there was an overrepresentation of cells at the outer edges of the scaffold. In a HE staining the repopulated scaffold tissue appeared as densely packed fibers with interspersed cells clearly visible by their cell nuclei (lilac), with a marked layer of cells lining the outer surfaces of the scaffold (Fig. 2A). The cell proliferation marker Ki-67 staining showed numerous proliferating cells at day 6, with few cells found at later time points (Fig. 2B). In contrast, there was no significant difference regarding the frequency of apoptotic cells, ranging from 1.0 to 6.2% in the individual sections (Fig. 2C). Immunofluorescent staining of the cultured scaffolds with the mesenchymal marker vimentin revealed intensively stained cells with elongated morphology, compatible with a well-preserved fibroblast phenotype (Fig. 2D). Image analysis showed that 30% of the cells were Ki-67 positive at day 6, with a significant decrease from day 6 to day 12 and day 25 (Fig. 2E). As a measure of the accumulated cell concentration in the scaffold we used a previously established proteomics method based on the summed MS intensities of the histones^41^. The summed MS intensities from the newly produced, heavy labeled histones for each time point demonstrated a non-significant increasing trend in accumulating cells, mirroring the proliferation measurements (Fig. 2F). The decrease in cell proliferation observed after day 6 is likely explained by cell-matrix contact inhibition or regulation by matrix components such as dermatan sulfate^42^. Possible variations in initial density of cells in individual scaffolds after seeding may influence the time until proliferation stops due to contact inhibition, and could explain some of the variation seen in Fig. 2E,F. In summary, the results showed that human lung tissue scaffolds support initial proliferation of primary human lung fibroblasts and their survival for at least 25 days in culture resulting in macroscopic reorganization of the tissue. The initial proliferative phase with an increase in cell numbers was followed by a non-proliferative phase with stable cell numbers after day 9.

### Matrisome composition of lung scaffold fibroblast cultures compared to monolayer fibroblast cultures

**Figure 3:**
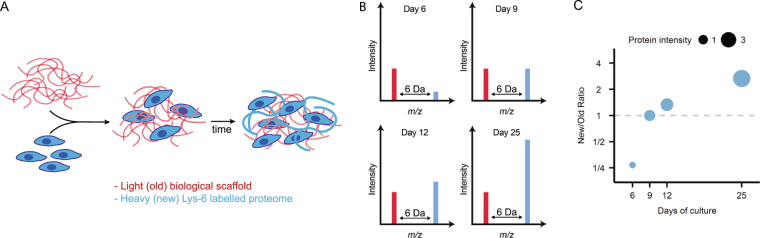
Stable isotope labeling of cells in native tissue scaffolds. (**A**) Decellularized scaffolds are seeded with cells and cultured in media containing heavy lysine (*Lys-6). All newly produced proteins contain heavy lysines (blue lines) whereas original scaffold proteins only contain non-labeled lysines (red lines). (**B**) Mass spectrometry analysis of the trypsin digested proteins produced doublet peaks separated by a discrete mass difference between labeled (blue bars) and non-labelled proteins (red-bars). The ratio between new peptides derived from newly synthesized proteins (new) and scaffold proteins (old) changes between time points as the tissue is remodeled. (**C**) A weighted scatter plot where a gradual accumulation of new protein is shown by an increase in point size with a corresponding shift in the ratio between new protein and old scaffold protein.

Fibroblasts are important producers of ECM and have been extensively studied in monolayer cell cultures. Considering the well documented bidirectional crosstalk between fibroblasts and surrounding ECM^6^, introduction of decellularized ECM scaffolds in fibroblast cultures will influence cell behavior. To quantify the influence of the scaffold-ECM on matrisome production, we cultured fibroblasts in lung scaffolds and in standard plastic culture dishes for up to 25 days. Importantly, the scaffold cultures were grown in heavy labeled media (SILAC) to differentiate between preexisting (old) proteins in the scaffold from new proteins produced by the fibroblasts (new) (Fig. 3). In this way, all newly produced proteins were shifted in mass and could be compared to the amount of preexisting proteins in the scaffold using mass spectrometry. This was then compared to the protein expression in standard monolayer cultures on plastic surfaces. Due to the dissimilar culture set-ups we could not accurately control for proliferative activity between the two systems. However, the monolayer cultures had reached confluency at 6 days of culture and proliferation in the scaffold cultures stopped between 6 and 9 days of culture, indicating that the proliferative phase were matched in the two culture set-ups and the comparison was made at a steady state at later time points. The number of identified proteins in native lung slices, repopulated lung slice scaffolds (after 25 days of culture) and monolayer cultures were 1211, 1609 and 2094, respectively (Supplementary Tables S2–S4).

**Figure 4:**
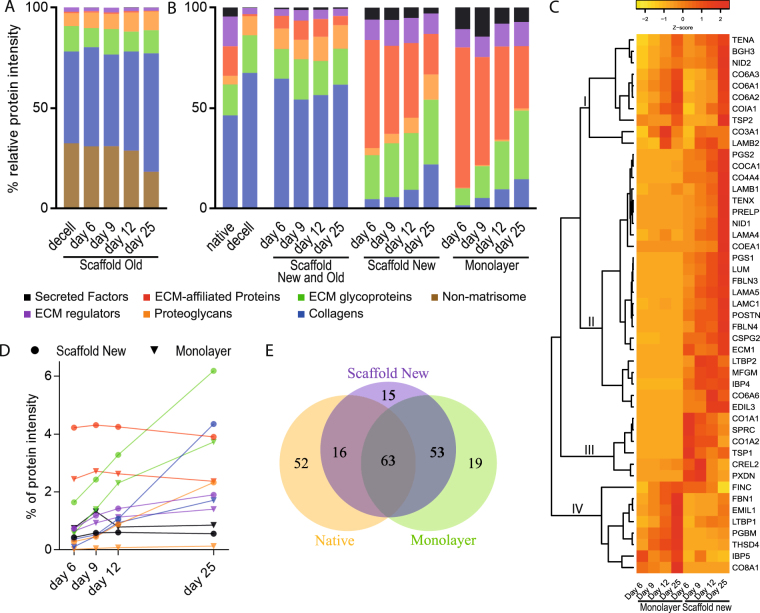
Fibroblasts repopulating tissue scaffolds modified the ECM and produced a different matrisome as compared to fibroblasts grown in monolayer culture. Cells were seeded on standard cell culture dishes or onto 3D scaffolds and cultured for up to 25 days followed by mass spectrometry analysis. (**A**) Relative contribution based on the summed protein intensities of individual matrisome groups from the original scaffold at different time points. (**B**) Relative MS-intensities for the six matrisome groups. First two bars show the native and decellularized lung tissue. The first set of four bars show the complete 3D tissue culture with both light and heavy signal (Scaffold Old and New). The two last sets of four bars show the newly synthesized matrisome proteins in the 3D scaffold cultures (Scaffold New) and the monolayer cultures respectively (Monolayer). (**C**) Heat map of core proteins for which protein intensities were registered at all four time points from either monolayer or 3D scaffold cultures, data scaled by row. Four sub clusters are marked in the figure, cluster I contains proteins with a similar abundance profile in monolayer and scaffold culture. II-III contains proteins abundant in 3D scaffold cultures but not in monolayer cultures. Cluster IV have proteins with a greater abundance in monolayer cultures. (**D**) Percentage of summed MS intensities of newly produced proteins subdivided into matrisome groups in scaffold and monolayer cultures over time. (**E**) Euler diagram comparing the number of matrisome proteins detected during tissue culture in the 3D scaffold compared to monolayer culture and native lung tissue.

Repopulation of the acellular scaffolds resulted in a 45% decrease of the old non-matrisome components over the 25-days culture period and an increase in the proportion of core matrisome proteins such as collagens and proteoglycans (Fig. 4A). In the native lung slices, prior to decellularization, 66% of the matrisome proteins were core matrisome proteins (blue, green and orange bars in Fig. 4B). As expected, decellularization efficiently removed the more loosely attached ECM associated matrisome proteins (lilac, red and black bars in Fig. 4B), elevating the proportion of core-matrisome proteins to 96% (Fig. 4B). The introduction of cells to the scaffold resulted in an initial increase in the proportion of ECM affiliated proteins, ECM regulators and secreted factors, as seen under “scaffold new” in Fig. 4B, indicating that the cells were replacing matrisome proteins that were depleted in the decellularization process. After nine days of tissue culture the relative amount of core matrisome protein started to increase. Collectively, the old and new proteins in the scaffold cultures made up a matrisome resembling the native tissue sections. This pattern showed that the added cells modified the matrisome towards a composition found in native tissue sections.

Production of core matrisome proteins by fibroblasts cultured in standard monolayers started off at low levels (monolayer in Fig. 4B) compared to the fibroblasts in scaffold cultures with little protein accumulated by day 6, but the proportion of core matrisome proteins increased over time in both settings. The most striking difference between the scaffold and the monolayer cultures was the low production of proteoglycans in the monolayer cultures, a difference that became more accentuated over time. To compare the ECM milieu facing the fibroblasts in the two culture settings we compared the combined matrisome of both old and new proteins in the scaffold cultures to the matrisome produced in monolayer cultures. In this comparison, the lower levels of core matrisome proteins in the monolayer cultures were even more striking, especially at the earlier time points (Fig. 4B). In addition to altering the relative composition of the matrisome proteins, the scaffolds promoted production of a higher proportion of matrisome proteins (Fig. 4B). At day 25, the matrisome proteins took up 19% of the new scaffold protein intensity compared to 11% in the monolayer cultures (data not shown). Apparent similarities between the culture settings were that ECM glycoproteins displayed the largest increase over time and the similar levels of ECM regulators and secreted factors in both culture settings (Fig. 4D). In contrast, there was a clear difference in levels of proteoglycans, which had close to a 25-fold higher proportional intensity (2.3% vs 0.1% total MS signal) in the scaffold culture at day 25 (Fig. 4D).

Individual matrisome proteins also showed different temporal expression patterns, which added to the distinction between the two cell culture settings. The heat map in Fig. 4C shows 47 core matrisome proteins that were identified at all four time points in either monolayer or scaffold culture. These core matrisome proteins are among the most abundant matrisome proteins and consequently reflects major aspects of extracellular matrix turnover. The unsupervised clustering generated four distinct sub clusters. The first cluster (I) of in total ten proteins contained three collagen VI-chains. Proteins in this cluster were associated with a similar temporal pattern in both culture settings with higher abundance at later time points, mirroring the increasing proportion of core matrisome proteins seen in Fig. 4B. Sub-cluster II-III contained proteins that were low abundant in the monolayer cultures, but had prominent abundance in scaffold cultures. Proteins in the larger cluster II increased markedly over time, e.g. the proteoglycans decorin, biglycan and lumican. Cluster III on the other hand contained proteins that were more abundant at earlier time points and showed diminishing signals as the culture time increased. Cluster IV contained proteins with a stronger signal in monolayer culture, the majority with a clear increase in protein levels over time. In total, the analysis identified 147 matrisome proteins synthesised in scaffold cultures compared to 135 in monolayer cultures, out of which 116 of these were shared between the two culture settings (Scaffold new and Monolayer in Fig. 4E). Overall, the fibroblasts in scaffold culture produced a matrisome with a slightly higher proportion of proteins in common with the native lung tissue 54% (79/147), compared to 47% (63/135) for fibroblasts in monolayer culture.

### Matrisome protein synthesis and turnover

**Figure 5:**
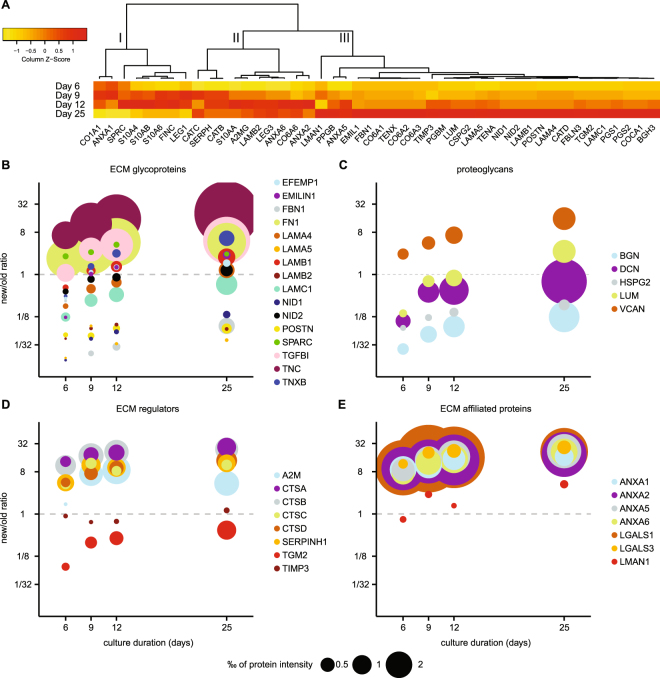
Matrisome protein turnover profiles in scaffold cultures. Mass spectrometric analysis of scaffolds repopulated with fibroblasts producing proteins containing stable isotope labeled amino acids. (**A**) Heat map showing the temporal profiles for matrisome proteins produced in scaffold culture (new), data scaled by column. Three sub clusters discussed in the text are marked with roman numerals. (**B**) Weighted scatter plot for ECM glycoproteins. Amount of newly synthesized proteins are represented by the size of the data points, ratio of new-to-old protein displayed on Y-axis with equal amount of new and old proteins at the dashed line. For schematic outline of the Figure type see Fig. 3B,C. (**C**) Weighted scatter plot for Proteoglycans, (**D**) ECM Regulators, (**E**) ECM-affiliated Proteins.

To further characterize ECM-remodeling during the first 25 days after repopulation of lung scaffolds with lung fibroblasts, we analyzed the remodeling pattern for individual matrisome proteins. We selected 46 matrisome proteins with both old and new protein intensity signals at all analyzed time points. The heat map in Fig. 5A shows the production of new protein for these 46 proteins, clustered according to how they increased in new protein intensities over time. From the selected 46 proteins, 27 proteins were confined in cluster III exhibiting a continuous increase of new protein over time. Proteins in this group were to a high degree core matrisome proteins with a structural extracellular function, among them proteoglycans and basement membrane components such as laminins, nidogens and perlecan (Fig. 5A). Most of these proteins (23/27) were also, as expected, associated with increasing new-to-old protein ratios as seen in the protein ratio heat map in Supplementary Fig. S1. Although 12 of the proteins still had a ratio of new-to-old proteins below 1 after 25 days of cultures (Fig. 5B–E, Supplementary Fig. S2, schematic outline of the Figure type shown in Fig. 3B,C). The pattern with a continuous production of new protein was especially apparent for the collagens (Supplementary Fig. S2A) and proteoglycans (Fig. 5C). These proteins were abundantly found in the decellularized scaffold and thus required considerable *de novo* synthesis or degradation of old protein to increase the new-to-old protein ratio above 1. In our analysis, the only proteoglycan with a new-to-old ratio >1 already at day 6 was versican, as shown in the bubble graph in Fig. 5C. The synthesis rate for all the proteoglycans followed an almost linear trend over the culture period, although for biglycan, perlecan and decorin the new-to-old protein ratios never reached above 1 after 25 days of culture (Fig. 5C).

In contrast to the consistent increase in proteoglycans over time, the proteins (*n* = 18) in cluster I and II in Fig. 5A peak in intensities around day 9–12. These protein production patterns mirror the decline in cell proliferation seen in the histologic evaluation (Fig. 2E), suggesting that some of these proteins might be of importance for initial repopulation of the scaffolds. These clusters contain several cell-associated proteins such as annexins, galectins, protein S100 and proteases such as cathepsins and peptidases. In addition, this group also included proteins such as fibronectin and collagen type VI, which are of importance in early ECM remodeling. In total 16/18 of these proteins reached a ratio >1 as many of these proteins were removed during the decellularization process (e.g. protein S100, Supplementary Fig. S2B) or were produced at a high rate in the beginning of the culture (e.g. fibronectin, Fig. 5B). One prominent pattern of this type can be found within the ECM regulators (Fig. 5D), which were removed during decellularization (Fig. 1F). Most of the ECM regulators have a high new to old ratio already at day 6, i.e. the newly produced proteins constitute the majority of the extracellular regulators present in the tissue. Five of the proteins are known to be intracellular; cathepsin B, cathepsin D, dipeptidyl peptidase 1 and lysosomal protective enzyme are all involved in lysosomal protease activity^43^, while serpin H1 is a proteinase inhibitor known to act as a chaperone in collagen maturation within the endoplasmic reticulum^44^. These five proteins followed a similar trend and have similar ratios at day 25 and the protein amounts appear to be linked to the cell content of the tissue. In the early phase of the cultures, proteins such as cathepsin B and D activate extracellular proteases, which together with the protease inhibitors alfa-2-macroglobulin and TIMP play important roles in the remodelling of the scaffold ECM (Fig. 5D, ECM regulators)^6^. Other proteins involved in the early changes of the scaffolds at day 6 and 9 are heavy labelled fibronectin, EMILIN-1 and Beta ig-h3, all of importance for cell adhesion, cell matrix interaction and ECM organization (Fig. 5B). Galectin-1 and several annexins (Fig. 5E, ECM affiliated proteins) are other newly synthesized proteins of early high expression, which are important for membrane organization and cell matrix interactions and cell proliferation.

Our results imply that the core-matrisome proteins are abundant in the decellularized tissues, but are still continuously produced by the fibroblasts. In contrast, matrisome proteins for which the relative amount of new protein does not continually increase tend to have a higher ratio at day 6–9 than those with a more positive trend. This may in part be explained by a more complete removal of these matrisome proteins in the decellularization process, but it may also indicate a role in initial remodeling of the tissue and differentiation of the repopulating cells. The results show that proteins that are produced in different amounts may still have a similar temporal pattern in ratio change, suggesting a similar turnover rate in the tissue cultures.

### Immunohistologic characterization of matrisome proteins

**Figure 6:**
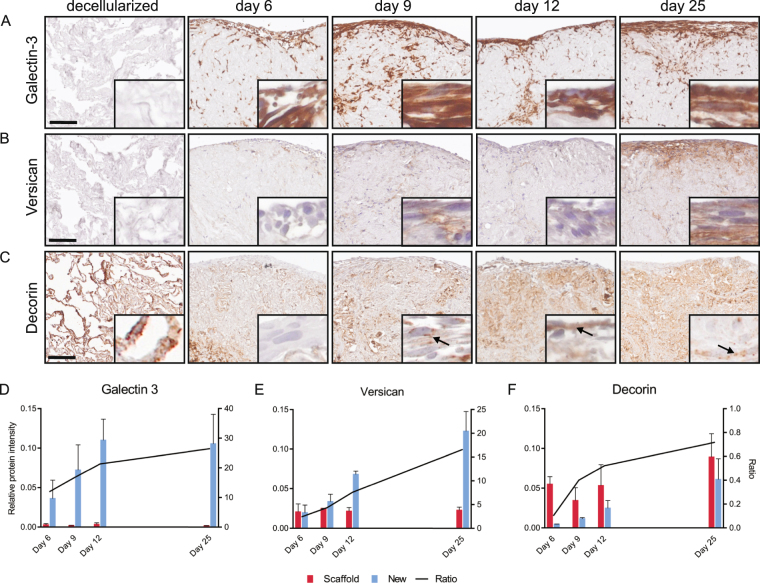
Immunohistochemical evaluation of selected proteins identified in the repopulated scaffolds. Immunohistological assessment of (**A**) galectin-3, (**B**) versican and (**C**) decorin, stained in decellularized and repopulated scaffolds at day 6, 9, 12 and 25. Scale bars:100 µm, inserts: x8 magnification of surface layer, arrows: pericellular decorin. Overview images of entire stained sections are presented in Supplementary Fig. S3. Bar graph of protein intensity for new proteins (blue), old proteins (red) and ratio (new-to-old, line) in repopulated tissues at day 6, 9, 12 and 25 for (**D**) galectin-3, (**E**) versican, (**F**) decorin.

Based on our findings of protein specific profiles of newly synthesized proteins, we performed IHC to evaluate if the quantitative differences corresponded to qualitative differences in the distribution of protein in the tissue. IHC evaluation was performed for three matrisome proteins selected based on differential temporal dynamics in the MS-data, having at least a 2-fold increase in new protein from day 6 to 25 and the availability of validated antibodies. Galectin-3 was the 27^th^ most abundant matrisome protein in our native lung slices, but was present only at low levels in decellularized scaffolds. Levels of new galectin-3 (Fig. 6A and D) increased rapidly during the first 12 days of tissue culture and then plateaued. In IHC staining galectin-3 was not visible in the decellularized scaffold, however after repopulation, a strong well delimited staining occurred around cellularized areas. This staining appears to be cell associated and the IHC and MS data for galectin-3 mirrors the increase in cells numbers (Fig. 2A,E and Supplementary Fig. S3A). This pattern may also be expected for other strongly cell associated matrisome proteins that are not extracellularly deposited, with tissue concentrations primarily reflecting the composition of cells in the tissue. Versican is found in the decellularized scaffolds at low levels and primarily found in interstitial matrix surrounding larger blood vessels and bronchioles (not included in the picture). New versican elevated almost linearly over the 25 days of tissue culture (Fig. 6B,E and Supplementary Fig. S3B). The IHC staining corroborates the increase over time. However, the versican staining pattern is different compared to galectin-3. Rather than being restricted to the cells as for galectin-3, the versican staining appeared as a gradient, with diminishing intensity further away from the cells, indicating extracellular deposition consistent with the known functions of versican^45^. The ratio of new-to-old decorin in the MS-data was below one at all measured time points, which is also reflected in the IHC where only a discrete increase in total staining was seen (Fig. 6C,F and Supplementary Fig. S3C). Decorin staining increased in repopulated areas of the scaffolds with a staining pattern indicating a pericellular distribution. The increase in new decorin signal displayed a similar trend to versican, but the histological change was subtle due to preexisting protein in the scaffold.

## Discussion

The understanding of ECM turnover in healthy and diseased lung tissue is hampered by incomplete knowledge of the balance between ECM production and degradation at a proteome-wide scale. In this work, we used a recently described SILAC-MS strategy to quantify for the first time the ECM turnover in acellular lung scaffolds repopulated with primary lung fibroblasts, grown in media supplemented with stable heavy isotope labelled amino acids. In this way, it was possible to quantify ECM turnover using mass spectrometry as this analysis can distinguish between residual ECM proteins found in the original scaffold from new ECM proteins produced by repopulating cells. Decellularization of the cryo-sectioned lung tissues resulted in removal of presumably loosely attached ECM associated matrisome proteins while the structural core of the ECM remained intact. After repopulation with cells, several of the ECM associated matrisome proteins were rapidly restored, mirroring the proliferative activity of the fibroblasts and indicating that levels of these proteins are linked to the number of cells in the cultures. In contrast, the acellular scaffolds promoted continuous accumulation of the core-matrisome proteins, most apparent for the proteoglycans. Collectively, the combined old and new proteins in the scaffold cultures resembled the matrisome found in the native tissue sections, indicating that the cells attempted to restore the ECM to a composition found in native tissue sections.

It is well documented that the surface that cells are cultured on influences cellular behaviour^46,47^. For example, decellularized ECM scaffolds contain surfaces supporting cell attachment and cell survival over time^48^. Additionally, scaffolds can control cell proliferation and supply biochemical and biomechanical cues to induce and/or maintain proper cell differentiation^48^. To quantify the effect of an existing matrisome on ECM turnover, we compared the matrisome production in standard monolayer culture with the matrisome production in acellular scaffolds. These experiments revealed that the acellular scaffold altered the matrisome production by increasing the proportional amount of core ECM proteins, most notably for the proteoglycans. The proportional increase of proteoglycans in the scaffolds may be a consequence of the pre-existing and organized 3D ECM structure promoting either increased production of proteoglycans or proper integration of the produced proteoglycans into the existing ECM^49^. In the monolayer cultures failure to incorporate the synthesized matrisome proteins may lead to secretion into the cell media, which was not analyzed in this experiment. Another explanation is that the presence of an existing ECM structure may support more efficient cell attachment, which could explain the delayed production of core matrisome proteins in the monolayer cultures.

In the scaffolds, the matrisome associated proteins are restored early during culture and displayed a new-to-old protein ratio higher than one. These proteins are typically intracellular and the majority of them plateaued in abundance level during the extended culture time. For example, the galectin-1 and galectin-3 MS protein intensities peaked around day 12. Histological evaluation of galectin-3 showed furthermore a delimited staining in cellularized areas, indicating that this protein is associated with the repopulating cells. Galectin-3 is a lectin involved in growth factor signaling for control of angiogenesis and fibrosis in several organs including lungs^50–52^. Inhibitors of galectin-3 is at present in a phase II clinical study for control of IPF^53^, where the mechanism is suggested to be a decreased expression of connective growth factor^54^. In contrast to the ECM associated proteins, the new-to-old ratio for all proteoglycans except versican was lower than one up to day 12. The accumulated amount of core-matrisome proteins increased continuously over time in the scaffold cultures and the pattern did not mirror proliferative activity observed in the scaffolds. These proteoglycans can all be expected to play a role in the remodeling of a scaffold by fibroblasts, by for example participating in collagen fiber assembly and organization^55^, supported by the production pattern of collagens that follows a similar trend. The distribution of versican and decorin obtained via IHC is different compared to galectin-3. Versican is a proteoglycan expressed by lung fibroblasts, involved in ECM remodeling in chronic obstructive pulmonary disease, asthma and bronchiolitis obliterans^45^. Decorin is a proteoglycan involved in matrix assembly and collagen fibrillation in particular, and together with its dermatan sulfate substituent it plays a regulatory role in growth factor signaling^11,55^. Decorin staining increased in repopulated areas of the scaffolds with a staining pattern indicating a pericellular distribution. Additionally, the continuous production of core-matrisome components over time in the scaffold cultures resulted in increasing level of collagen I and VI. The latter is an important component in the basement membrane and for the formation of protective network around the fibroblasts^56^.

We show on a proteome wide scale how the presence of an existing lung ECM structure influence protein synthesis, but it remains unknown how different ECM structures and combination of cells affect ECM turnover. A study by Li *et al*. used SILAC-MS to quantify ECM turnover using vocal fold mucosa repopulated with previously characterized vocal fold fibroblasts^25^. In line with the study by Li *et al*., our results showed a rapid synthesis of ECM glycoproteins at the early time points. One prominent example of this is fibronectin, which was massively produced early on. Fibronectin plays a role in cell attachment and represents an important molecule for cells to attach firmly to the scaffolds^57^. We detected several laminins, which are part of a family of glycoproteins crucial for basement membrane function^58^, that were not found in the Li *et al*. study. This might in part be explained by the greater proteomic depth in our study as we identified more than twice the number of proteins (Supplementary Fig. S4). As for the proteoglycans and collagens, we found an increasing trend throughout the extended culturing time, also in line with the Li *et al*. study^25^. However, some discrepancies in the type of proteoglycans and collagens were evident, and strikingly we showed that more ECM proteins reached a new-to-old protein ratio higher than one, despite only half the culture time compared to the study of Li *et al*. This was especially apparent for the ECM glycoproteins and can in part be explained by the lower starting concentration of ECM glycoproteins in our lung scaffolds. It might also indicate a more rapid repopulation and ECM turnover rate in our system. In addition, other differences in experimental conditions such as cell numbers may play a role.

In this work, we used MS-data to select matrisome proteins for IHC evaluation and the results from these two methodologies are in good agreement for the selected proteins. The ability to differentiate between newly produced ECM proteins from residual proteins in lung scaffolds from cryosections of human lung tissue provides new information on how ECM is remodeled in the lung. In this study we used fibroblasts, being main effector cells for ECM production and wound repair. Co-culture with other cells normally residing in lung parenchyma, such as pneumocytes, alveolar macrophages, endothelial cells and mesenchymal stem cells will likely modify the ECM production and turnover. The method presented here represents a valuable technique to further evaluate the contribution of other cell types, or combination of cell types, during these processes. The application of SILAC-MS to scaffold systems from healthy or diseased lung tissue will advance our understanding of ECM repair and remodeling by revealing how different cell types produce and degrade ECM depending on the composition of the underlying scaffold.

## Electronic supplementary material

Supplementary Figures and Table S1

Supplementary Table S2

Supplementary Table S3

Supplementary Table S4
