# Supplementary material for: Quantifying extracellular matrix turnover in human lung scaffold cultures
Source: Sci Rep. 2018 Apr 3;8:5409. doi: 10.1038/s41598-018-23702-x (PMC5882971; doi:10.1038/s41598-018-23702-x)
Supplement: Supplementary file 1 — Supplementary Figures and Table S1 [file 41598_2018_23702_MOESM1_ESM.pdf]

Quantifying extracellular matrix turnover in human lung scaffold cultures

Oskar Rosmark[1,*], Emma Åhrman[1,2], Catharina Müller[1], Linda Elowsson Rendin[1], Leif Eriksson[1], Anders Malmström[1], Oskar Hallgren[1,3], Anna-Karin Larsson-Callerfelt[1], Gunilla Westergren-Thorsson[1,†], Johan Malmström[2,†]

[1] Lung Biology, Dept. Experimental Medical Science, Lund University, Sweden

[2] Division of Infection Medicine, Dept. Clinical Sciences, Lund University, Sweden

[3] Dept. Respiratory Medicine and Allergology, Lund University, Sweden

* Corresponding author. *E-mail address*: oskar.rosmark@med.lu.se

†these authors contributed equally to this work

Supplementary Material:

Supplementary Figure S1

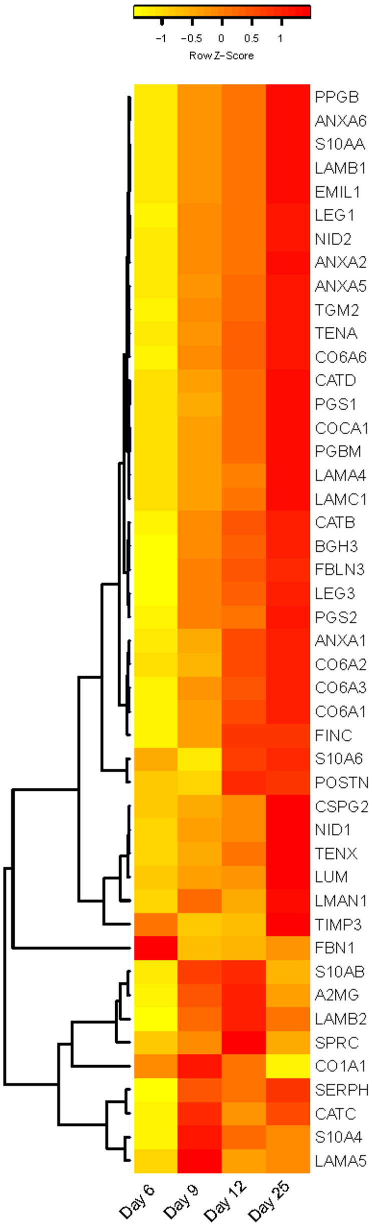

Supplementary Figure S1. New-to-old ratios for matrisome proteins in scaffold cultures. Heat map showing the temporal profiles for the ratios between matrisome proteins produced in scaffold culture (new) to the proteins originally present in the scaffold (old), data scaled by row.

Supplementary Figure S2

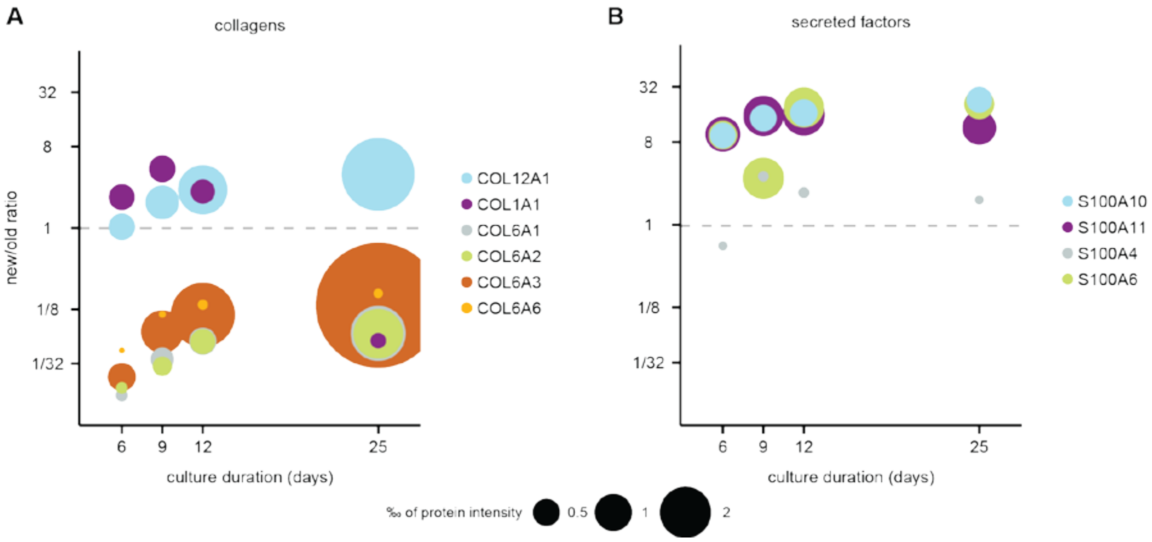

Supplementary Figure S2. Additional matrisome protein turnover profiles in scaffold cultures. Weighted scatter plots of (**A**) collagens and (**B**) secreted factors. Amount of newly synthesized proteins are represented by the size of the data points, ratio of new to old protein displayed on Y-axis with equal amount of new and old proteins at the dashed line.

Supplementary Figure S3

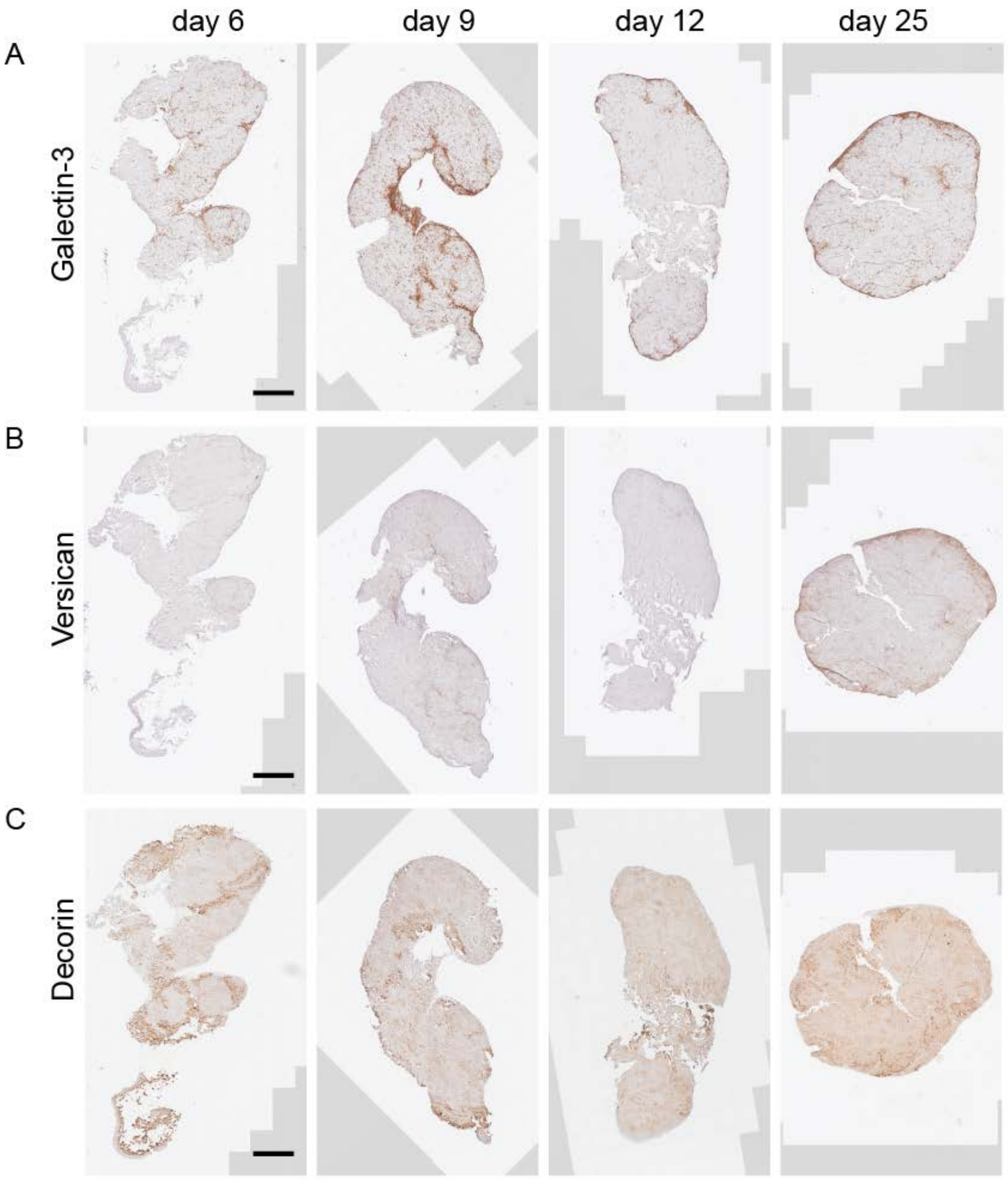

Supplementary Figure S3. Overview images of whole immunohistologically stained tissue sections. Representative magnified images are seen in Figure 6. Staining for (**A**) galectin-3, (**B**) versican and (**C**) decorin. Positive staining in brown. Scale bar 500 µm. The pictures visualize the compaction of the scaffolds over time by the fibroblasts.

Supplementary Figure S4

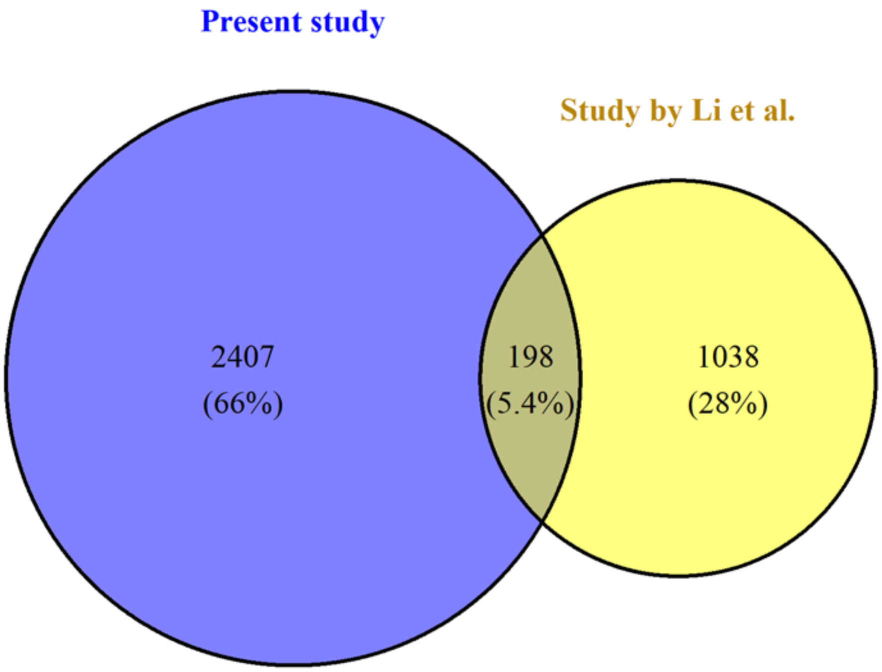

Supplementary Figure S4. Identified protein in our data compared to analogous study with scaffold from vocal fold mucosa by Li et al*. Euler diagram showing number of proteins identified by mass spectrometry in repopulated scaffolds that is shared between and unique for the two data sets.

* Q. Li, Z. Chang, G. Oliveira, M. Xiong, L.M. Smith, B.L. Frey, N. V. Welham, Protein turnover during in vitro tissue engineering, Biomaterials. 81 (2016) 104–113.

Supplementary Table S1.

Primary antibody	Manufacturer	Antigen retrieval	Dilution	Secondary antibody
Collagen type IV	Abcam, Cambridge, MA, US, ab6586	heat mediated: EnVision™ FLEX Target Retrieval Solution, Low pH	1:1000	Alexa Fluor® 647 (Invitrogen, Eugene, OR, US)
Ki-67	Abcam, ab15580	heat mediated: EnVision™ FLEX Target Retrieval Solution, Low pH	1:1000	Alexa Fluor® 647 (Invitrogen, Eugene, OR, US)
Vimentin	R&D, AF2105	heat mediated: EnVision™ FLEX Target Retrieval Solution, Low pH	1:100	Alexa Fluor® 555 (Invitrogen, Eugene, OR, US)
Versican	Atlas Antibodies, Uppsala, Sweden HPA004726	Chondroitiase ABC*	1:750	Envision, Dako K4065
Galectin-3	monoclonal, hybridoma supernatant†, kind gift from Prof. Hakon Leffler[#]	Chondroitiase ABC*	1:50	Goat anti-rat HRP Sigma A9037
Decorin	Atlas Antibodies, Uppsala, Sweden HPA003315	heat mediated: EnVision™ FLEX Target Retrieval Solution, High pH	1:1000	Envision, Dako K4065

*0,025 U/ml chondroitinase ABC in special TBS (0.1 M Tris, 0.3 M NaCl, ph 7.6) for 30 min at 37°C

[#] Dept. for Microbiology, Immunology and Glycobiology, University of Lund, Sweden

† M.K. Ho, T.A. Springer, Mac-2, a novel 32,000 Mr mouse macrophage subpopulation-specific antigen defined by monoclonal antibodies., J. Immunol. 128 (1982) 1221–8.
